# Supercharged End-to-Side Nerve Transfer for Ulnar Neuropathy: Redefining Nomenclature and Recommendations for Standardisation of Surgical Technique Description

**DOI:** 10.7759/cureus.48660

**Published:** 2023-11-11

**Authors:** Chane Kulenkampff, Liron S Duraku, Samuel George, Dominic Power

**Affiliations:** 1 Peripheral Nerve Surgery Department, University Hospitals Birmingham NHS Foundation Trust, Birmingham, GBR; 2 Department of Plastic, Reconstructive and Hand Surgery, Amsterdam University Medical Centers, Amsterdam, NLD

**Keywords:** anterior interosseous nerve to ulnar nerve transfer, nomenclature, standardisation, surgical technique, ulnar nerve neuropathy, reverse end to side nerve transfer, supercharged end to side nerve transfer

## Abstract

Supercharged end-to-side (SETS) nerve transfers have been described as a treatment option for ulnar neuropathy, however, there is inconsistency in the nomenclature used to describe the microsurgical technique. The purpose of this article is to systematically review the available literature on the SETS nerve transfer technique and to provide an overview of the technical variations to facilitate standardisation of surgical method.

A literature review was performed through PubMed, MEDLINE, and Ovid databases according to the Preferred Reporting Items for Systematic Reviews and Meta-Analyses (PRISMA) guidelines. Studies that reported surgical technique of anterior interosseous nerve (AIN) to ulnar nerve SETS transfer were included. Studies were excluded when not referencing SETS/reverse end-to-side (RETS) nerve transfers, studies referencing nerve transfers other than AIN to motor fascicle bundle of the ulnar nerve (MUN), animal studies, and studies not reporting technique.

Of the 168 studies found, 14 articles were included. In five articles, distal visualisation of the MUN in Guyon’s canal was specifically cited. In the four studies that commented on donor preparation, sharp neurectomy proximal to the AIN branching point was undertaken. Recipient preparation was commented on in seven of the included studies. Two studies referred to an epineurial window only while five specifically recommended a perineurial window. Coaptation site was specified in four studies and all studies used sutures for coaptation, with four articles stipulating that 9-0 nylon was used. Additionally, fibrin glue was used in conjunction with suture technique in four studies.

Consistency in nomenclature used to describe SETS microsurgical technique is needed before case series measuring outcome can be reliably interpreted. This review allowed for the development of suggestions for standardisation of nomenclature and minimal reporting requirements when describing SETS technique*. *Standardisation of technique will allow for reproducibility and facilitate future evaluations of outcome in prospective randomised control trials.

## Introduction and background

Supercharged end-to-side (SETS) nerve transfers have been described as a treatment option for ulnar neuropathy since first performed in humans in 2009 [1]. In recent years there has been an increase in publications on the topic, however, there appears to be inconsistency in the nomenclature used to describe the relevant anatomy and the microsurgical technique. With SETS transfers growing in popularity, it is now necessary to obtain consistency in nomenclature and description of technique which will allow for future evaluation of outcomes in prospective randomised control trials (RCTs). 

There is discrepancy surrounding the terms used to describe the SETS transfer with this term first being coined the name reverse end-to-side (RETS) nerve transfer in 2005 and later being referred to as SETS by Barbour et al. in 2012, with variations of this term including “supercharged”, “supercharge” and “supercharging” [1]. There is also much inconsistency in the reporting of the technique in the literature with several articles not commenting on key components of the procedure. In the initial approach and planning phase extension into Guyon’s canal to “visually neurolyse” the deep motor fascicle bundle of the ulnar nerve (MUN) is commonly considered in practice; however, the exclusion or inclusion of this extension is not universally commented on in articles describing technique.

From a microsurgical technique approach there again seems to be little consensus. The level of the donor nerve harvest, as well as its preparation prior to coaptation is described in poorly defined detail, with inconsistency between written reports and referenced educational videos. In addition, there is variation in recipient nerve dissection when considering epineurial window size and depth of dissection within the MUN with some studies advocating for inclusion of a perineurial window rather than epineurial window opening alone. This is likely due to inappropriate use of the term “perineurial” with lack of recognition of the interfascicular epineurium when describing depth of surgical technique. Most sources indicate the coaptation site relative to the volar wrist crease, however, the topography for the MUN is rarely considered when describing the site of coaptation. Suture technique is also infrequently described in appropriate detail which would preferably include whether the use of fibrin glue was used in the repair.

The aim of this study is to provide a review of the available literature on SETS nerve transfer technique and to offer an overview of the technical nuances to facilitate standardisation of nomenclature used and to provide a recommendation on the minimal reporting criteria to use when describing SETS technique.

## Review

Methods

Literature Search

A critical review of all the available literature on SETS technique was undertaken. The Preferred Reporting Items for Systematic Reviews and Meta-Analyses (PRISMA) guidelines were followed when conducting this review. A search was undertaken of PubMed, MEDLINE, and Ovid databases. Keywords searched were: Supercharged-End-to-Side (including variations supercharge and supercharging), Reverse-End-to-Side, and anterior interosseous to ulnar nerve transfer. The search was conducted in September 2022, for studies published in any year. In addition, article reference lists were reviewed to identify addition any publications that may have been missed by the primary search.

Study Selection

**Figure 1:**
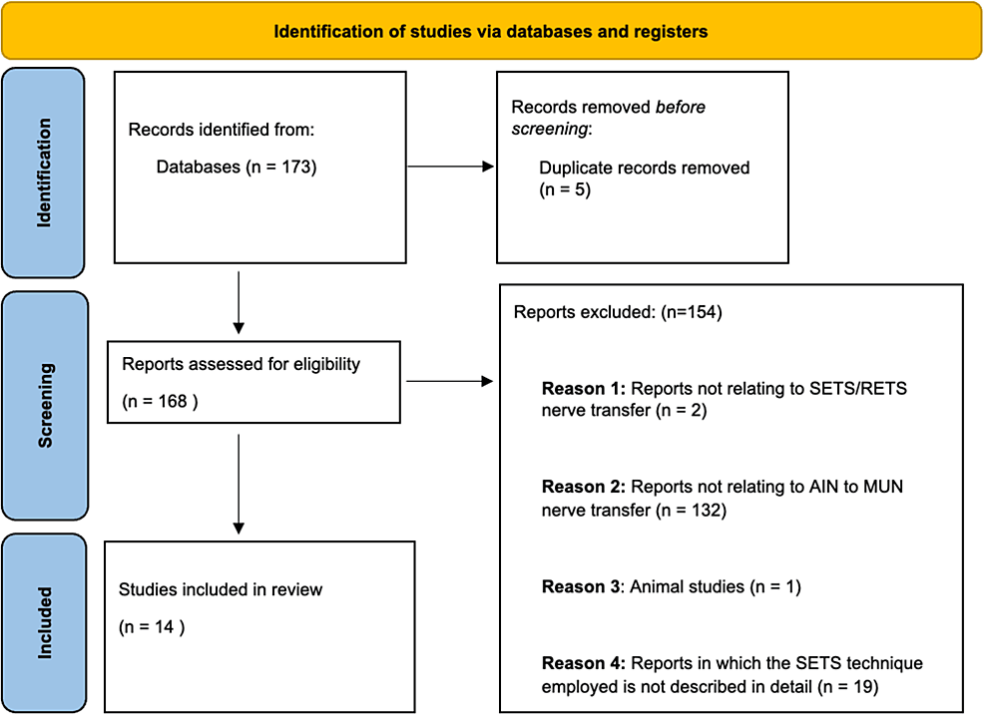
Flowchart of the literature screening according to PRISMA guidelines PRISMA: Preferred Reporting Items for Systematic Reviews and Meta-Analyses, SETS: Supercharged end-to-side, RETS: Reverse end-to-side, AIN: Anterior interosseous nerve, MUN: Motor fascicle bundle of the ulnar nerve

Articles meeting the following criteria were included: studies reporting surgical technique of anterior Interosseous nerve (AIN) to MUN SETS transfer. Duplicates were removed. Studies were excluded when not referencing SETS/RETS nerve transfers, animal studies and studies referencing nerve transfers other than AIN to MUN and studies not reporting technique. Figure 1 demonstrates the flowchart of the literature screening.

Results

Study Selection

The literature search identified 173 studies; after removing duplicates, a total of 168 studies remained. After screening of abstracts, 14 were eligible for inclusion. These are included in Table 1.

**Table 1:** Included studies demonstrating description of surgical technique AIN: Anterior interosseous nerve, MUN: Motor fascicle bundle of the ulnar nerve, FDI: First dorsal interossei, DBUN: Dorsal branch of the ulnar nerve, FPB: Flexor pollicis brevis, PQ: Pronator quadratus

Title	Date	Authors	Study type	Technical description		Comments	
Distal Anterior Interosseous Nerve Transfer to the Deep Motor Branch of the Ulnar Nerve for Reconstruction of High Ulnar Nerve Injuries	2002	CB Novak et al. [2]	Case series	Donor preparation	AIN divided proximal to branching point		
Distal median to ulnar nerve transfer to restore ulnar motor and sensory function within the hand: technical nuances	2009	J Brown et al. [3]	Technical description	MUN identified in Guyon’s canal	Visual neurolysis from Guyon’s	Taleisnik incision recommended	
Supercharged End-to-Side Anterior Interosseous to Ulnar Motor Nerve Transfer for Intrinsic Musculature Reinnervation	2012	J Barbour et al. [1]	Technical description	MUN identified in Guyon’s canal	√		
				Recipient preparation	Epineurial & Perineurial window (5mm)		
				Suture use	√ 9-0		
				Fibrin glue use	√		
Anatomical and histomorphometric observations on the transfer of the anterior interosseous nerve to the deep branch of the ulnar nerve	2015	TL Schenck et al. [4]	Cadaveric study	MUN identified in Guyon’s canal	√ Visual neurolysis	“The poor ratio of AIN to MUN could be addressed by transferring the AIN to selected DBUN fascicles that are expected to be most helpful for hand function”	
				Coaptation site	Suggest specific fascicle group targeted		
Supercharged End-to-Side Nerve Transfer: Too Soon for “Prime Time”?	2012	J Isaacs [5]	Letter to editor	Recipient preparation	Epineurial window only	“A window through the connective tissue around the motor group of fascicles (which is epineurial tissue, not perineurial tissue) in the human ulnar nerve will reveal a group of fascicles. Each fascicle is surrounded by perineurium, and a perineurial window in this situation would only get you into 1 fascicle.”	
							“Too soon for prime time”: In Reply to Dr Isaacs	2013	S Mackinnon [5]	Letter to editor	Donor preparation	Fan out the distal portion of the AIN	“The AIN divides into 3 branches at the midpoint of the pronator quadratus muscle, so theoretically, separate repairs could be done. However, we do not think this is necessary if the perineurium is opened widely and the AIN is fanned out to maximally cover the motor branch .“	
Recipient preparation	Epineurial & Perineurial window (Open widely)												
Refining Indications for the Supercharge End-to-Side Anterior Interosseous to Ulnar Motor Nerve Transfer in Cubital Tunnel Syndrome	2019	HA Power et al. [6]	Case series and description	Donor preparation	AIN is divided as it branches in the midportion of the muscle	“The anterior interosseous nerve fascicles are splayed across the motor fascicles”								
				Recipient preparation	Epineurial (2 to 3mm) & Perineurial window (2 to 3mm)								
				Coaptation site	9cm proximal to the wrist.		
				Suture use	9-0 nylon		
				Fibrin glue use	√		
Interfascicular Anatomy of the Motor Branch of the Ulnar Nerve: A Cadaveric Study	2021	SB Chambers et al. [7]	Cadaveric study	Coaptation site	9cm proximal to the wrist.	Demonstrates internal topography of ulnar nerve motor fascicles. Refers to Brown et al. technique where the AIN is coapted to the radial side of the ulnar nerve where the FDI and FPB are located.	
Reverse End-to-Side (Supercharging) Nerve Transfer: Conceptualization, Validation, and Translation	2021	J Isaacs et al. [8]	Descriptive	Recipient preparation	Epineurial window only (2 to 3mm)	“Manipulating the fascicles within to create perineurial windows and suturing to the edge of the epineurial window would be challenging for the novice microsurgeon”	
Outcomes of anterior interosseous nerve transfer to restore intrinsic muscle function after high ulnar nerve injury	2021	SC George et al. [9]	Case series and SR	Recipient preparation	Epineurial & Perineurial window (2 to 3mm)		
				Suture use	9-0 nylon		
				Fibrin glue use	√		
Mechanisms and outcomes of the supercharged end-to-side nerve transfer: a review of preclinical and clinical studies	2021	N von Guionneau et al. [10]	Descriptive review	Suture use	9-0 nylon		
				Fibrin glue use	√		
Five Reliable Nerve Transfers for the Treatment of Isolated Upper Extremity Nerve Injuries	2021	BR Peters et al. [11]	Descriptive	MUN identified in Guyon’s canal	√	“The epineurial window is used to “tuck” the anterior interosseous nerve fascicles”	
				Recipient preparation	Perineurial window (at least 5mm)		
				Coaptation site	6 to 7cm proximal to the wrist crease		
				Suture use	Sutures are placed through the epineurium		
Reliability of deep branch of ulnar nerve identification in interosseous-to-ulnar motor nerve transfer: A cadaver study of 20 wrists	2021	S Moling et al. [12]	Cadaveric study	MUN identified in Guyon’s canal	√ Guyon’s canal release is advisable	“Without identification in Guyon’s canal the DBUN was incorrectly identified in 10% of cases.”	
Ulnar nerve decompression and transposition with versus without supercharged end-to-side motor nerve transfer for advanced cubital tunnel syndrome: a randomized comparison study	2022	Q Xie et al. [13]	A randomized comparison study	Donor preparation	AIN divided at proximal edge of PQ		

Of the 14 included studies, two were letters to the editor, five were descriptive studies, three cadaveric studies and two case series, one systematic review and one randomized comparison study. 

In five of the studies, distal visualization of the MUN in Guyon’s canal was explicitly mentioned. Moling et al. looked specifically at the necessity of this additional incision and found in their cadaveric study of 20 wrists that the deep branch of the ulnar nerve was incorrectly identified in 10% of cases when no Guyon’s canal identification was undertaken [12].

Donor preparation technique was also assessed in the literature. In the four studies that commented on this, sharp neurectomy proximal to the AIN branching point was done and no surgeons suggested that a more distal division was necessary. In one paper, this was elaborated to describe a “fan out” technique for preparing the donor nerve, however, in a later open-source educational video describing the technique, the same author demonstrated dissection of individual AIN branches that are then brought together and coapted through a single epineurial window [5,14].

Recipient preparation was commented on in seven of the included studies. Two studies referred to an epineurial window only, while five specifically recommended a perineurial window. The epineurial/perineurial window size described in most reports was a 2-3mm window. No studies reference coapting individual branches through multiple epineurial windows, however this was described as being theoretically possible in Mackinon’s letter referenced above [14].

Coaptation site was specified in four studies, two of these suggested a coaptation at 9cm proximal to the wrist crease and one was a little more distal at 6 to 7cm. The fourth study by Schenck et al commented specifically on the precise fascicles that should be targeted when deciding coaptation site, taking into consideration the reliable topography of the ulnar nerve and the poor ratio of AIN to MUN [4]. Given this, he recommends that coaptation to fascicles that are expected to be most helpful for hand function should be undertaken [4].

All studies used sutures for coaptation, with four stipulating that 9-0 nylon was used. Fibrin glue was used in conjunction with suture technique in four studies.

Discussion

The results of our literature review show inconsistency in reporting of technique in terms of distal visualization in Guyon’s canal, donor preparation, recipient preparation, coaptation site, suture technique as well as fibrin glue use. The purpose of the discussion which follows is to suggest a standardized use of nomenclature and minimal reporting requirements that authors should consider using when describing SETs technique. This is of particular importance in outcome studies where an alternation from standard technique may have a direct impact on outcome.

The Nomenclature

**Figure 2:**
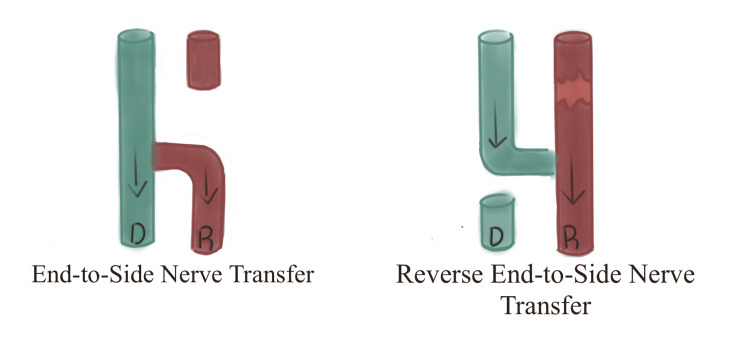
Schematic diagram of end-to-side nerve transfer and SETS/RETS nerve transfer. D: Donor nerve in green, R: Recipient nerve in red. Arrows show the direction of innervation. SETS: Supercharged end-to-side, RETS: Reverse end-to-side

Before a discussion can be had on the microsurgical technique, the correct use of nomenclature and anatomical definitions needs to be established. The first is that there is no consensus as to the term used to describe the technique of coapting the transected donor nerve to the side of the recipient nerve to allow for neural regeneration distally. Originally this was described as reverse end-to-side nerve transfer and this term is still commonly used even in recent work. This chosen name builds on from the end-to-side (ETS) transfer where the distal end of a recipient nerve is sectioned and coapted to the side of a functioning donor nerve [15]. This difference is illustrated in Figure 2.

The terms, however, have proven to be confusing as it is in conflict with the standard way in which surgeons describe the direction of flow in the surgical context. This point is echoed in Dellon et al.’s 2010 paper entitled “Which end is up” where he makes the comparison to a free flap which has a donor site and goes to a recipient site and elaborates that this same concept can be seen in neural regeneration which proceeds from proximal to distal [16]. Thus, the use of the term RETS is arguably not the most appropriate option. The term supercharged end-to-side later coined by Barbour et al. seems a more appropriate choice as it alludes to the concept that this technique adds additional axons to the neural regeneration process [1]. To minimize confusion and to allow for standardization, the authors recommend the use of “supercharged end-to-side transfer” when referring to this technique going forward, a recommendation supported by von Guionneau et al. [10].

**Figure 3:**
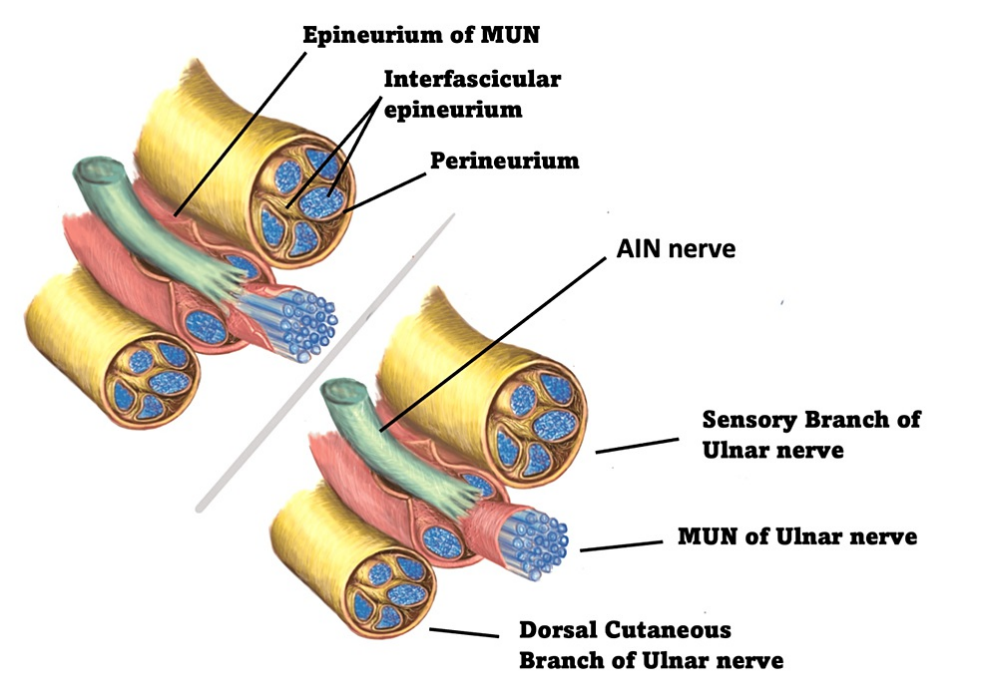
Illustration of ulnar and AIN nerve anatomy in SETS technique The illustration shows a cross section of the ulnar nerve in the forearm as a sensory-motor-sensory sandwich, the loose interfascicular epineurium is shown within and between the sensory and motor fascicle groups. SETS: Supercharged end-to-side, AIN: Anterior interosseous nerve, MUN: Motor fascicle bundle of the ulnar nerve

Delving further into the nomenclature used to illustrate SETS it is important to ensure that the anatomy is properly defined. It is generally accepted that the epineurium represents the outer anatomical border of an individual nerve [17]. The perineurium on the other hand is composed of circumferential layers of flattened cells around the nerve trunk within the epineurium [18]. Reina et al. in their work using tissue-specific staining, have demonstrated well that there is an additional, less commonly cited collagen layer that underlies the epineurium and surrounds the perineurium which they refer to as “internal epineurium” a term which is used synonymously with the name “interfascicular epineurium” within the article. The perineurium should therefore not be confused with the interfascicular epineurium, which, unlike the perineurium, contains no cells [18]. Figure 3 illustrates this anatomy.

When a SETS transfer is performed it is not possible to reliably distinguish the perineurium from the interfascicular epineurium in the hands of the novice microsurgeon and it is possible that published case reports are only achieving dissection through the interfascicular epineurium rather than through the perineurium as they describe it. This sentiment is supported by Jonathan Isaacs in his 2012 letter to the editor- “Too soon for prime time”, where he states that the illustration drawn in the technique guide of Barbour et al. is inaccurate, as a window through the connective tissue around the motor group of fascicles would be through epineurial not perineurial tissue [1,18]. Isaacs and others make reference to the fact that there is weak evidence that a perineurial window may allow greater donor axon regeneration into the recipient nerve and perineurial dissection will potentially expose and damage axons creating a risk of downgrading recipient nerve function in the continuity nerve lesion [10]. The optimal window depth should balance the hypothesized benefits of improved donor axon penetration with the risks of impacting native regeneration [10]. Finally, the depth of perineurium dissection cannot be reliably controlled and, therefore, the interfascicular epineurium approach is arguably both safer and easier to standardise.

A final point on the relevant anatomy to be considered in an AIN to MUN SETS transfer is brought to light by Chambers et al. in their 2021 work looking at the interfascicular anatomy of the MUN in cadaveric samples [7]. Their research demonstrated a predictable arrangement of the motor fascicles in all specimens. The arrangement from radial to ulnar was as follows: flexor pollicis brevis, first dorsal interossei (FDI)/intrinsic muscles and abductor digiti minimi (ADM). Knowledge of this anatomy may have implications when considering the SETS technique coaptation site in selected patients. This research would also suggest that the concept of a hemi end-to-end (ETE) transfer has no role in AIN to MUN transfer, as this would only achieve recovery in half of the muscle groups and this would effectively be an ETE transfer to these specific motor fascicles.

The Macrosurgical Approach

The standard approach includes an incision in the palm for identification of the motor branch in Guyon’s canal through performing a Guyon’s canal decompression, with an extension into the forearm. The recommendation from Mackinnon who first performed the AIN to MUN in clinical practice, is to “visually neurolyse” the motor from the sensory fascicle group at this level and trace this back to the standard coaptation site, just distal to the dorsal cutaneous branch take off at 6-8cm proximal to the wrist crease. The inclusion of a distal Guyon’s canal release for identification of the MUN is further supported by Moling et al. [12] in their cadaveric study which demonstrated that 10% of MUN fascicular groups were incorrectly identified when a Guyon’s release was not performed.

The Microsurgical Approach

A SETS nerve transfer for ulnar neuropathy involves coaptating the cut end of the AIN to the side of the MUN. Options for donor nerve preparation can be broadly divided into three techniques. In addition, different options for recipient nerve preparation have been described with other specifications including suture or suture-less repair alternatives.

Variables Surrounding the Donor Nerve Harvest and Coaptation

In the original clinical description of SETS for AIN to MUN transfer by Barbour et al. the donor AIN is harvested using sharp neurotomy proximal to its branching point [1]. Mackinnon elaborates on this in her response letter to Isaacs describing that fascicles of the cut end of the AIN can be “fanned out” prior to coaptation [5,7].

An alternative to this technique includes dissection of individual nerve branches distally and coaptating branches individually to the side of the MUN through multiple epineurial windows. This technique is not widely described in the literature except for a mention in MacKinnon’s letter that it is theoretically possible, but likely unnecessary if the perineurium is opened widely while incorporating the technique of fanning out the AIN. One must ensure with this technique that the articular branch of the AIN (distal to pronator quadratus (PQ)) - a purely sensory branch is not used in the SETS transfer as this will not precipitate the intended benefit [5]. The advantages of dissecting out the individual branches include increased length of donor nerve, which ensures a tension free repair. It is necessary that this potential gain is weighed against the risk that this may require additional sutures for coaptation with the potential for damage to the small donor branches.

**Figure 4:**
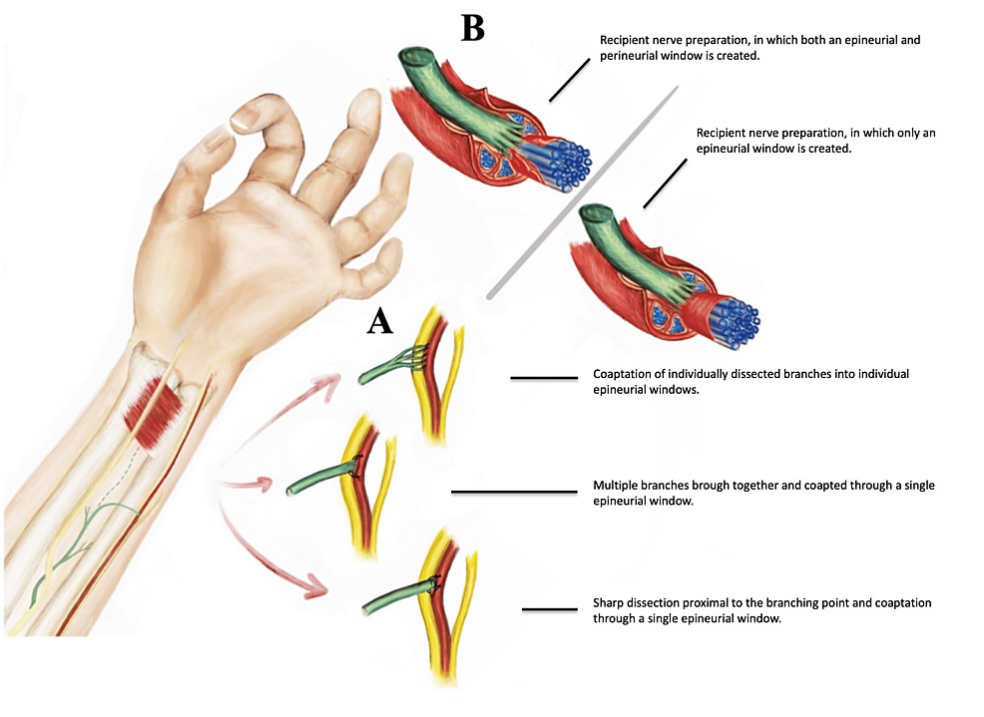
Illustration showing donor and recipient nerve preparation and options for microsurgical coaptation Illustration A shows the microsurgical repair options from top to bottom including: Coaptation of individually dissected branches, multiple branches brought together and coapted through a single epineurial window, and lastly an illustration of sharp dissection proximal to the branching point and coaptation through a single epineurial window. Illustration B shows two options of recipient nerve preparation, the image on the left indicates dissection to beyond the perineurium, and the right most image shows preservation of perineurium with breaching of the epineurial window only.

Lastly there remains the option of bringing branches together through one epineurial window as can be seen in MacKinnon’s open-source educational video [14]. This final option maximises length of donor but facilitates a simpler single coaptation site. This technique is, however, limited by the length of the shortest (most proximal) PQ branch. Figure 4 illustrates the neural anatomy and microsurgical technique variations.

Variables Surrounding the Recipient Nerve Preparation

The anatomical level of dissection through and beyond the epineurium is not standardised with some surgeons advocating for coaptation through an epineurial window alone while others specify that scoring of the perineurium is required for axonal propagation. Much of the research refers to scoring the perineurium when, in fact, it may be that a true perineurial breach is not commonly achieved and a plane of dissection within the interfascicular epineurium is all that is achieved. Opening the perineurium risks direct injury to axons within their tubes such that effectively an ETE connection may be facilitated with distal endoneurial tubes being repopulated by the growing AIN axons, at the expense of the native axon. The inability to assess the degrees of recipient axon injury and depth of interfascicle dissection render this approach risky and unpredictable. Opening of the perineurium is not controlled and therefore difficult to reproduce.

The size of the epineurial and perineurial window is also not agreed upon and recommendations vary anywhere from 2 to 5mm in recent publications by Walker et al. [19].

A proposal for a 5mm window was made by Walker et al. in their work on end-to-side neurotomy in rats, suggesting in this study that this allows for greater collateral sprouting and regenerative response without increasing donor nerve morbidity [19]. 

The site of coaptation to the MUN, whether this be radial/midline or ulnar is not specifically mentioned in most articles but may hold clinical significance based on the work by Chambers et al. referenced earlier [7]. Standardisation of this coaptation site is something that may be considered based on patient presenting features.

Variables Surrounding Suture vs Suture-Less Coaptation

Currently there is no agreed number, depth or technique of suture placement widely recommended for the coaptation. A 9-0 suture size was used consistently where this was referenced in the included papers.

Sutured repair technique standardly involves simple repair from one edge of the recipient epineurial window to the donor nerve epineurium. An alternative to this is a suture repair that incorporates both sides of the epineurial window in an attempt to adequately close the window around the donor nerve with the hypothesis that this may help to prevent axonal escape. This technique, while not commonly described in the literature, is routinely practiced in some institutions, including our own. A disadvantage of this technique includes the potential to constrict the nerve and impair the blood-nerve barrier

The additional use of a fibrin glue is also widely suggested. There is evidence to suggest that use of fibrin glue alone may be sufficient and has shown success in situations where tension needs to be mitigated such as nerve gap reconstruction with nerve grafts [20]. Results from this and other work on the topic appear promising, though more work is needed in this field before it may be used to inform recommendations for standardisation. Detail on this research is beyond the scope of this paper and opportunities for refinement with suture-less techniques and polymer glues/hydrogels should be explored in the future.

Suggested Minimal Reporting Requirements When Describing Technique

For documentation of technique in future publications, the authors suggest the following standardisation of technique along with minimal reporting requirements in Table 2 that would allow for consistency in future reporting.

**Table 2:** Standardisation of Technique SETS: Supercharged end-to-side, AIN: Anterior interosseous nerve, MUN: Motor fascicle bundle of the ulnar nerve

Standardisation of technique	Minimal reporting requirements
Step 1 Identify the MUN fascicle group in Guyon’s canal	Specify if MUN identified in Guyon’s canal.
Step 2 Harvest AIN	Document if donor (AIN) neurotomy performed proximal or distal to branching point
Step 3 Identify location of the SETS coaptation	Document location of SETS coaptation proximal to wrist crease
Step 4 Create MUN epineurial window (5mm)	Document epineurial window size and if perineurium breached.
Step 5 Trim the end of the donor AIN, do not remove any donor nerve epineurium	Commentary on preparation of donor end
Step 6 Insert donor nerve 2-3mm into the interfascicular epineurium of MUN	Depth of insertion into interfascicular epineurium to be recorded. Record tension of repair.
Step 7 Suture Close the epineurial window. Suggest 2 x 9-0 nylon sutures placed in non-constricting manor, incorporating both sides of epineurial window	Description size and number of sutures used and if both sides of epineurial window incorporated in suture. Record non-constricting repair.
Step 8 Seal the nerve repair with fibrin glue	Document use of fibrin glue +/- conduit / wrap support

## Conclusions

The literature on the SETS technique is limited as descriptions are inadequate, anatomical terms are used inconsistently and key components of the procedure are not detailed.

Consistency in nomenclature used to describe SETS microsurgical technique is needed before reports measuring outcome can be reliably interpreted. Standardisation of technique will allow for reproducibility and facilitate future evaluations of outcome in prospective randomised control trials.
